# Attitudinal Barriers Hindering Adoption of Telepsychiatry among Mental Healthcare Professionals: Israel as a Case-Study

**DOI:** 10.3390/ijerph182312540

**Published:** 2021-11-28

**Authors:** Tamir Magal, Maya Negev, Hanoch Kaphzan

**Affiliations:** 1 School of Public Health, University of Haifa, Haifa 3498838, Israel; mnegev@univ.haifa.ac.il; 2 Department of Neurobiology, Faculty of Natural Sciences, University of Haifa, Haifa 3498838, Israel; hkaphzan@univ.haifa.ac.il

**Keywords:** telepsychiatry, telemedicine, tele-mental health, e-mental health, internet-based intervention, efficiency, professional experience, health maintenance organizations, health knowledge, attitudes, practice patterns, video-assisted treatment, organizational culture

## Abstract

Despite proven advantages for the use of telemedicine in psychiatry, mental healthcare professionals have shown deep-seated mistrust and suspicion of telepsychiatry, which hinders its widespread application. The current study examines the attitudes of Israeli mental health professionals towards telepsychiatry and seeks to uncover the effects of experience and organizational affiliation on its adoption. The methodology included qualitative and thematic analysis of 27 in-depth interviews with Israeli mental health professionals, focusing on three major themes—clinical quality, economic efficiency, and the effects on the work–life balance of healthcare professionals. The attitudes of mental health professionals were found to be widely divergent and sharply dichotomized regarding different aspects of telepsychiatry and its suitability for mental healthcare services. However, there was a general consensus that telemedicine may not fulfil its promise of being a panacea to the problems of modern public medicine. In addition, attitudes were related to hierarchical position, organizational affiliation, and personal experience with telepsychiatry. Specifically, organizational affiliation influenced experience with and support for the assimilation of telepsychiatry. The study also revealed the role of organizational leadership and culture in promoting or inhibiting the proliferation and adoption of innovative technologies and services in modern medicine.

## 1. Introduction

Telemedicine has become increasingly popular in medical practice and in academic research, especially so following the outbreak of COVID-19. This is partially because of its distinct ability to overcome barriers of time and space and its ability to bring diverse and advanced medical services to remote and rural areas [1,2,3,4,5,6,7]. Previous research highlights several advantages of telepsychiatry, especially in regard to increased accessibility and ease of services for patients [4,8,9,10,11]. However, a major obstacle for the spread and assimilation of telepsychiatry into traditional mental healthcare services has been the attitudes, perspectives, and emotional responses of healthcare professionals to this new technology [12,13,14]. The aim of this study is to examine the attitudes towards telepsychiatry of mental health professionals in Israel’s public healthcare system and the relationship between their individual experience with telepsychiatry, organizational affiliation, and support for telepsychiatry.

### Background

Academic research on telemedicine and telepsychiatry has experienced a significant surge in publications over the last 30 years [1,8,15,16]. Here, we focus on the attitudes of healthcare professional towards telepsychiatry in three distinct aspects: efficiency and economic benefits, quality of therapeutic treatment, and the effects on healthcare professionals. Empirical research has shown the economic advantages of telepsychiatry for both clients and therapists [9,10,11]. Studies have also demonstrated the economic cost-effectiveness of telepsychiatry for Health Maintenance Organizations (HMOs) and service providers [17]. Additionally, some studies have suggested that the use of telepsychiatry might reduce the phenomena of late- and no-shows as well as shorten actual session-time, thereby reducing congestion and alleviating the bottleneck of wait-times [8]. In terms of the quality of treatment, comparative studies have shown telepsychiatry to be non-inferior and essentially non-distinguishable from conventional psychiatric treatment [18,19,20,21,22].

However, studies examining the experiences and attitudes towards telepsychiatry have demonstrated a significant gap between healthcare professionals and the general population. These studies reveal deep-seated levels of concern, suspicion, and distrust in telepsychiatry among therapists and psychiatrists, which is not shared by their clients [11,12,13,14,23]. Additionally, numerus studies have shown technical problems to be a major concern among mental healthcare professionals, hindering their adoption of telepsychiatry [9,11,12,24]. Therefore, the current study will seek to describe and examine the opinions and attitudes of Israeli therapists and psychiatrists towards the efficiency, applicability, and quality of telepsychiatry in the context of Israel’s public healthcare system.

## 2. Materials and Methods

### 2.1. The Israeli Context

Israel has a universal and compulsory public healthcare system, in which every citizen in insured from birth until death and is entitled to a basic level of healthcare services. Healthcare services are provided by four public HMOs (herein named HMO A, B, C, D), which compete over customer enrollment and satisfaction and are reimbursed for services that are rendered by the government [25,26].

Since 2015, mental healthcare services are provided by the HMOs. Additionally, there are several government-owned regional clinics as well as general and psychiatric hospitals that are able to provide emergency and long-term treatment. In addition, many Israeli psychiatrists and other MDs supplement their day job in the public healthcare system with a private clinic in the afternoons [26,27].

Telemedicine technologies have been gradually introduced into the Israeli public healthcare system over the last decade. Significant differences were observed in the rate and depth of implementation between various healthcare providers. The field of mental healthcare has been a late comer to this innovative process, with the introduction of online child psychiatry consultations being introduced in 2016 [28]. Telepsychology and telepsychiatry services for adults were initiated by some HMOs in 2019 [29], with online social workers and emergency services being added in 2020 due to the outbreak of COVID-19 pandemic [30,31].

### 2.2. Study Design

A qualitative study consisting of in-depth interviews with mental healthcare practitioners in the Israeli public healthcare system, including senior- and mid-level professional managers.

### 2.3. Recruitment of Participants

After identifying over a hundred HMO- and government-owned local clinics as well as psychiatric and general hospitals within the public mental-healthcare system, potential interviewees were contacted by phone and e-mail. The research population consisted of psychiatrists working within the public healthcare system. Participants were selected on the basis of purposive sampling quotas [32,33], which were designed to reflect the various healthcare organizations (all HMOS and government), organizational hierarchy levels (national managers, regional managers, hospital managers and local clinics), and geographical diversity (National, North, Center, South) (see Table 1). Numerous appeals were made to the regional and local clinics of all four HMOs in each geographical region of the country, where individual psychiatrists were invited to take part in this study. We contacted 68 individual psychiatrists, of which 27 agreed to participate in an interview. Due to its qualitative nature and small sample size, the current study is not representative of all Israeli mental healthcare professionals. However, such a sampling strategy enhances the diversity of the participants along a regional, hierarchical, and organizational basis.

### 2.4. Data Collection and Analysis

Individual in-depth semi-structured interviews were conducted with each participant in a location of his/her choosing. The guidelines for these interviews were developed after a thorough review of the literature on telepsychiatry and included open-ended questions and prompts regarding the themes and issues that emerged from the literature. The interviewees were questioned regarding their perceptions and attitudes towards different aspects of telepsychiatry as well as their actual familiarity and experience with it (see Appendix A for the interview protocol). Interviews were conducted by the first author (PhD, male, with significant past experience and training in qualitative interviewing) between 10th December 2019 and 10th March 2020. At the beginning of each interview, the interviewer explained the research goals, and the interviewees signed an “informed consent” form. A typical interview lasted for 45–60 min, was digitally recorded, and was later transcribed for analysis. The authors had no prior acquaintance with the interviewees and no further relationship following the interview.

These transcripts underwent inductive thematic analysis according to the principles of grounded theory analysis [34,35]. Analysis was conducted through Qualitative-Data Analysis software (ATLAS.ti, v.8, ATLAS.ti Scientific Software Development GmbH, Berlin, Germany). Thematic consistency was achieved through intercoder reliability checks for several interviews by two independent coders (ICR = 0.82). Throughout the analytic process, the authors met regularly to discuss and compare preliminary findings. As a result of these discussions, the initial codes were grouped and merged into categories and themes. Finally, three major themes emerged, which were then reviewed to ensure internal consistency and coherence.

Furthermore, in order to assess the effects of antecedent experiential and organizational factors on attitudes towards telepsychiatry, the authors created two combined scales-representing one’s “support for-” and “experience with telepsychiatry”. The authors quantified participants’ positions on specific aspects of telepsychiatry along a dichotomy of “support/oppose” and then aggregated these results to create a “support for telepsychiatry” scale. It is important to emphasize that this scale is only used for illustrative purposes and not intended for statistical analysis. Experiential knowledge of telepsychiatry was also similarly quantified using information provided during the interviews. Then, after grouping participants according to their organizational affiliation, the study compared them along their experience with and support for telepsychiatry.

## 3. Results

### 3.1. Participants’ Characteristics

A total of 27 participants were interviewed for the study (40% response rate). They represent a cross-sectional and diverse sample of the public mental healthcare system in terms of both organizational affiliations and hierarchical positions. Table 1 presents further details regarding their characteristics.

### 3.2. Interview Findings

The thematic analysis yielded the following three themes of attitudes towards telepsychiatry: (a) quality of treatment using telepsychiatry, (b) efficiency and economic advantages associated with telepsychiatry, and (c) effects on the professional and personal lives of psychiatrists. In the following, we present each theme and provide quotes from the interviews. Finally, we analyze the relationship between organizational affiliation, experiential knowledge, and support for telepsychiatry services.

#### 3.2.1. Quality of Treatment Using Telepsychiatry

When asked about the effects of telepsychiatry on the quality of psychiatric examination and treatment, approximately half of the interviewees felt that the quality of treatment was as good as face-to-face, while the other half thought it to be inferior. In other words, there was no clear consensus on the matter. For example, about half of our respondents expressed their opposition to online examination and diagnosis, emphasizing its inferiority and inadequacy compared with traditional face to face examination. These respondents referred to one’s inability to gather non-verbal and pre-interview cues, such as movement, smell, body language, disquiet, etc. Additionally, they referred to lack of unmediated and intimate acquaintance with the patient, which they said would negatively affect interpersonal contact and therapeutic alliance. A regional manager for an HMO explained:

*In a preliminary meeting with a patient, there is paramount importance to direct interpersonal interaction and body language. There are lots of senses, sensors, and skills that need to be employed in order to formulate a preliminary assessment of the patient—and the online medium is very restrictive in that sense. [Interviewee 13]*

Some interviewees also referred to the social role of face-to-face therapy sessions—forcing the patient to exit his house, mingle with other people, and spending some quality time in meaningful conversation with their therapist. They expressed concern that the use of videoconferencing would all but isolate these patients, cutting their social ties and “releasing” them of the “burden” of social interactions. A senior psychiatrist in a general hospital emphasized:

*With some of my patients, the most significant improvement in their situation stems from the fact that they need to get up in the morning, leave their home, take the bus, and interact with other people on their way to the clinic. From my perspective, there is a significant therapeutic value in leaving one’s home… I fear we will lose that significant something when interacting through a screen [telepsychiatry]. [Interviewee 26]*

By contrast, a significant group among our respondents expressed their confidence in their ability to perform a proper examination online and issue a recommendation for the involuntary commitment of a patient following such examination. In a similar vein, these respondents also perceived tele-psychiatric treatment to be clinically comparable to conventional face-to-face treatment. For example, a psychiatrist from the northern region expressed the opinion that:

*There are no limitations on conducting a proper psychiatric examination in telepsychiatry. I do not conduct a slopier or less thorough examination online. My personal feeling is that there is no impairment on my ability to diagnose a patient via telepsychiatry. [Interviewee 04]*

In conclusion, the participants in this study were evenly divided in their estimation of the quality of treatment with telepsychiatry. No clear consensus was found regarding the question of clinical comparability of telepsychiatry with traditional face to face treatment or its effect on therapeutic alliance. Some of the participants expressed their concerns regarding its effects on increased social alienation in patients.

#### 3.2.2. Efficiency and Economic Benefits

The interviewees referred to two aspects of efficiency and economic benefits: for the patients and for the HMOs. Regarding the patients, the vast majority of our interviewees referred to the efficiency and economic benefits of telepsychiatry in terms of accessibility, travel costs, commute times, and loss of daily income. A regional manager in northern Israel phrased this succinctly:

*The most significant advantage in telepsychiatry is in reducing the costs for patients—in travel costs, commute time, and loss of wages. [Interviewee 08]*

However, concerning the advantages for the HMOs in terms of reducing no-shows, wait times, or economic costs, our interviewees were far less conclusive and expressed evenly divided opinions. With regard to economic costs, about half of the respondents estimated that HMOs will indeed be able to benefit from lower expenditure on office space, but this may be rolled on to the independent psychiatrists and online therapists, who will have to bear the costs of a private clinic or of working from home. By contrast, the other half of the interviewees estimated that HMOs will increase their expenditure on office-space rentals in order to accommodate both patients and psychiatrists, who are now present at different locations. These divergent views can be partially predicated based on organizational affiliation, as some HMOs have enacted stringent limitations on telemedicine through private computers and cellphones. A senior manager for one of the HMOs explained:

*In reality, telepsychiatry is more expensive for HMOs because you need to occupy two rooms at the same time—one for the doctor and another for the patient. This means double rental and maintenance costs. [Interviewee 02]*

Additional economic benefits associated with telepsychiatry included the possibility of lower payment to the psychiatrist for conducting an online treatment compared to face-to-face. While viewed as an advantage for the HMO, many interviewees perceived such possibility as frugality at their own expense. A therapist from a local HMO clinic put it bluntly:

*The HMO is paying less for an online therapist, compared to what they pay a regular therapist, or even in comparison to an independent/freelance one. [Interviewee 17]*

Another efficiency and economic issue that was raised in the interviews is the high no-show rate in current face-to-face treatment, which results in both wasting the therapists’ time and in longer waiting times for patients. About half of our respondents claimed that telepsychiatry would not solve the problem of no-shows. They thought that it would be very difficult to fill up vacancies with short notice (less than 3 h), even with the availability of virtual waiting lists. A senior psychiatrist in a general hospital remarked:

*From our experience, the percentage of no-shows in telepsychiatry is very similar to traditional treatment. People treat it as no different from a regular appointment. The usage of virtual waiting rooms might reduce this rate somewhat. But, it’s something we have yet to try. [Interviewee 23]*

About two-thirds of the interviewees referred to the issue of long wait-times between referral and appointment. About half of them believed that the ease and accessibility of telepsychiatry would create additional demand for mental health services. Which, in turn, would negate and even roll back any improvement in wait times. The lead psychiatrist in a regional clinic in Israel’s center succinctly explained:

*In my opinion, even if it works wonderfully and without malfunctions, telepsychiatry will only serve to extend wait times because of a well-known rule in health economy: the more efficient and accessible a service is, the higher its demand and usage. [Interviewee 22]*

The respondents also referred to the possibility of utilizing telepsychiatry to shorten the length of therapeutic sessions, thereby enabling the reception of more patients per hour. A majority among them opposed the possibility of shortened sessions either because it was perceived to be professionally inappropriate or because of expected technical difficulties. Head of a department in a psychiatric hospital remarked:

*In my opinion, a session of less than 30 min is not a “real” and meaningful session… it’s not even long enough in order to maintain rapport and build a working alliance. [Interviewee 16]*

Taken together, these examples illustrate the duality of the participants’ perspectives regarding the efficiency of telepsychiatry. A vast majority of the participants concurred that telemedicine is very economical and efficient from the patients’ point of view. However, the participants were vehemently divided regarding its efficiency for the HMOs or for the therapists themselves. Many of them expressed concerns that the HMOs would roll additional costs onto the therapists in order to ensure economic efficiency.

#### 3.2.3. Effects on Therapists

The participants in this study were evenly divided in their assessment of the effects of telepsychiatry on the work–life balance of therapists. While many interviewees acknowledged the advantages of telepsychiatry, others raised concerns regarding its effects on the work environment of psychiatrists and therapists. A sizable group of our interviewees adamantly objected to the possibility of extending work hours in telepsychiatry to the late afternoon and evening time, referring to the need for private life, the maintenance of work–life balance, as well as the need to supplement their income through private practice. One such therapist, from a psychiatric hospital in the center of Israel, clearly stated:

*My wife will not be happy if I’ll be closed-off in the study from 19:00 to 22:00, videoconferencing with patients, instead of spending time with my children. It will be very damaging to the work–life balance, which we strive so hard to preserve. [Interviewee 16]*

Many of the participants emphasized the difficulty of maintaining a proper and dignified setting for their online sessions, referring to household disturbances or poor location choices by their patients. Household disturbances refer to phone calls, children, delivery people, or pets suddenly appearing in the frame. Poor location choices refer to the places where the patients choose to conduct the session from. The most commonly sighted improper locations include bedrooms, bathrooms, and cars as well as the patient’s workplace.

A significant portion of the interviewees sighted technical difficulties associated with telepsychiatry—such as with Internet/network connectivity or with the operability of the software employed—as a major obstacle that undermines the very possibility of conducting regular psychiatric sessions online. A regional clinic manager from Israel’s center expressed frustration:

*It’s very difficult to overcome the technical issues involved [with telepsychiatry]. The conversation is interrupted regularly, and it takes a few minutes to resume connection. A significant portion of session-time is wasted on connectivity issues. Even afterwards, the sound quality is poor, and it is hard to hear the patient. [Interviewee 22]*

While most participants concurred that telepsychiatry would dramatically reduce the risk of physical violence against therapists, some of our interviewees referred to increases in “negative social behavior” by their online clients. These phenomena—such as rudeness, impatience, and “window shopping” between therapists—are also prevalent among offline patients. However, these participants perceived a correlation between the ease and comfort of online telepsychiatry and the spread of these phenomena. A senior psychiatrist form the Jerusalem area put it succinctly:

*Convenience [of telepsychiatry] is all well and good, as long as it’s available to the patients... However, the moment there is a problem, and this level of convenience and service they’ve gotten used to is no longer available, from a verity of reasons, this raises the level of frustration and anger, which may lead to aggressiveness and even violence. [Interviewee 13]*

In summary, the participants raised significant concerns regarding the effects of telepsychiatry usage on the work environment of mental healthcare professionals. These concerns included technical and logistical difficulties that affected the ease and flow of communication, increases in negative behavior by patients, as well as effects on the therapists’ work–life balance.

### 3.3. Relationship between Organizational Affiliation, Experiential Knowledge and Support for Telepsychiatry Services

**Figure 1:**
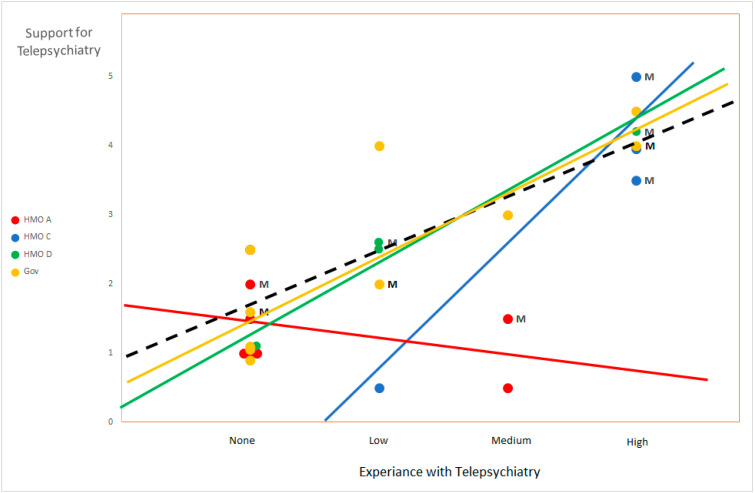
Interviewees’ experience with- and support for telepsychiatry.

Finally, we sought to examine the relationship between attitudes towards telepsychiatry and personal experience with this method of treatment. Figure 1 presents a correlation of the participants’ experience with and support for telepsychiatry. Observations are color-coded according to organizational affiliation. The lines added to the figure represent trend lines for each organization (fully color-coded lines) as well as a trend line for the entire sample (dashed black line). The letter “M” designates participants in management positions.

We found organizational differences in support for telepsychiatry as well as correlations between experience and support for telepsychiatry (see Figure 1). Participants from HMOs C and D as well as government employees exhibited a positive correlation between experiential knowledge of and support for telepsychiatry. Additionally, our findings show that participants in managerial positions, who are generally more experienced in telepsychiatry, exhibited a more positive approach towards telepsychiatry. In two of the four HMO’s (C and D), these managers were also instrumental in introducing telepsychiatry into their organizations in the first place.

By contrast, the employees of HMO A showed low levels of support for telepsychiatry, regardless of experiential knowledge or management role. The trend line for HMO A reveals a slightly negative correlation between experience and support for telepsychiatry. These results may be indicative of a negative organizational atmosphere towards telepsychiatry, or a poor online infrastructure and technical support in HMO A, which enjoys an extensive and widespread infrastructure of local clinics throughout Israel. However, it is important to remember that, due to the small sample size and the biased nature of the sampling technique, such findings should be seen as inconclusive in nature.

Another interesting finding is the lack of participation by HMO B employees. Despite numerous inquiries and recuring invitations, only one therapist from HMO B participated in this study. Several other “potential participants” from this HMO attributed their refusal to “lack of authorization by superiors”. We hypothesize that this avoidance/refusal to participate might be indicative of an organizational atmosphere of opposition to or insecurity regarding telepsychiatry.

## 4. Discussion

### 4.1. Principle Findings

The goals of the present study were to explore the attitudes and perceptions of mental healthcare professionals towards the usage of telepsychiatry. Relying on previous research [10,17,19,22], we anticipated that these professionals would acknowledge the clinical equivalence of telepsychiatry and the economic efficiency, together with apprehensive attitudes towards its effects on practitioners. We found that the Israeli psychiatric community is strongly divided in regard to the efficiency and usage of telepsychiatry. Our participants were evenly divided between supporters and opponents of telepsychiatry and its widespread usage in routine mental healthcare. This division manifested itself most clearly in their assessment of the clinical quality of telepsychiatry. However, it was also palpable in their assessment of its efficiency and effects on healthcare professionals.

Overall, the present study revealed a positive correlation between the participants’ experiential knowledge with telepsychiatry and their support for its usage. However, this correlation seems to be mitigated by one’s employment and organizational affiliations. Therefore, while most of our participants exhibited a similar correlational trendline indicating that higher experience with telepsychiatry leads to higher support for its usage, The employees of a single HMO (A) exhibited a contradictory and negative trendline. For these participants, more experience with telepsychiatry also meant stronger opposition for its utilization. These findings may indicate that while a positive correlation is the norm, a conducive organizational climate and hierarchical support are necessary conditions for the assimilation and proliferation of telemedicine. (See [36,37,38,39] for similar findings in other fields of medical innovation).

### 4.2. Comparison with Prior Works

Previous studies have demonstrated clinician concerns regarding the quality of online examination as well as regarding therapeutic alliance during telepsychiatry [12,40,41]. Others emphasized the technical difficulties and limitations that are associated with online communication [9,10,11,42]. A few studies even mentioned therapist concerns regarding the potentially increased social avoidance of their patients [41,42,43]. However, for the most part, these findings were sidelined in their final conclusions while emphasizing overall satisfaction. The present study focused specifically on these issues, emphasizing and expanding our understanding regarding these concerns.

Furthermore, while some studies referred to the effects of experience with telepsychiatry and its relationship with support for online treatment [12,44], these findings were mostly anecdotal and non-systematic. The present study contributes to the literature in this regard by conducting a systematic and rigorous correlation between the participants’ attitudes towards and experience with telepsychiatry, in which we found that, in general, experience with telepsychiatry led to higher support. Exceptions may be related to specific organizations, which we suggest may be related to their organizational climate or to the quality of online services that are provided for caretakers and patients.

The critical role of managers and opinion leaders in the adoption and implementation of telemedicine services has been emphasized by several studies comparing telemedicine implementation between different providers [12,42,45,46,47]. These studies are congruent with our findings regarding the differences between HMOs in attitudes towards telepsychiatry and support our hypothesis regarding the role of organizational hierarchy, atmosphere, or infrastructure in explaining these differences. Specifically, in HMOs where management policies are supportive of telepsychiatry, the attitudes of the professionals were more favorably inclined towards it. By contrast, when management was reserved or hesitant towards tele-mental health treatment, the workers’ attitudes tended to be negatively inclined.

The incursion of telemedicine and telepsychiatry on the work–life balance and personal space of practitioners was not, thus far, an issue of interest in telemedicine research (see a rare exception: [48]). We found this to be a significant concern among clinicians, who worried about the incursion of extended working hours on their private lives. Our findings highlight the need for further research regarding these concerns and suitable means for supporting clinicians in maintaining work–life balance while providing tele-services, for example, through a more flexible time-management of their workload or assistance with bureaucratic work.

### 4.3. Limitations

A small sample size, selection bias, and lack of generalization are among the most common limitations associated with the implementation of qualitative research methods. In the present study, the authors attempted to rectify some of these limitations by employing regional and organizational quotas for participant selection to ensure diversity and representation. However, the sample size and generalization still remained an issue. Furthermore, the application of a quasi-statistical method and language in the second part of this article is meant purely for illustrative purposes and should not mislead readers to attribute any statistical power to our findings. Future research should aspire to further examine the relationship between experiential knowledge and support for telepsychiatry, utilizing more robust statistical methods.

## 5. Conclusions

Our findings show that attitudes towards telepsychiatry are dependent on the position of interviewees within the organizational hierarchy as well as on their experience with telepsychiatry. That is, psychiatrists in managerial roles who are generally also more experienced with tele-mental health services exhibited a more positive approach towards telepsychiatry. As was shown in this study, the attitudes of mental health professionals are divergent and often dichotomized regarding different aspects of telepsychiatry and its suitability for the Israeli public mental healthcare services. At the same time, these attitudes are related to one’s hierarchical position, to the distinct institution, and to his/her personal experience with telepsychiatry. Nonetheless, personal experience is influenced by the institution’s attitude towards telepsychiatry, which determines the support in its assimilation. The mental health professionals who participated in this study were in consensus that telemedicine may not fulfil its promise of being a panacea to the problems of modern public medicine. Although qualitative in nature, our findings are also relevant to other countries that provide mental health services through a public health system, including Western and Central European countries. Importantly, practitioners’ experience with telepsychiatry is related to positive attitudes towards this service and may help overcome prejudice towards online mental health treatment. This can help healthcare systems to provide better mental healthcare services in times of high demand, such as during crisis or epidemic as well as in rural and remote regions, which often suffer inequality and neglect in access to healthcare services.

The study was conducted shortly before the COVID-19 pandemic and therefore provides a snapshot of the pre-COVID situation. This pandemic accelerated the introduction of telemedicine into all fields of medicine, including telepsychiatry. The current situation emphasizes the importance of research on telepsychiatry in order to overcome barriers and to provide improved mental health services. The study also revealed the role of organizational leadership and culture in promoting the proliferation and adoption of innovative technologies and services in modern medicine. Future research should further examine the role of hands-on experience and institutional culture in the proliferation of innovative health technologies and services, and examine the perceptions of patients regarding telepsychiatry.

## Figures and Tables

**Table 1:** Distribution of interviewees.

		Hierarchical Position			
		National Managers	Regional Managers	Hospital Managers	Local Clinics
**Healthcare organizations**	**HMO A**	1	2	3	2
	**HMO B**	1			
	**HMO C**	1	2		1
	**HMO D**	1	2		1
	**Gov**	1		4	5
**Total**		27			
**Geographical regions**	**National**	5			
	**North**		2	2	3
	**Center**		2	3	4
	**South**		2	2	2
**Total**		27			

## Data Availability

The data presented in this study are available upon request from the corresponding author. The data are not publicly available due to confidentiality and anonymity considerations. The excerpts used in the article were deducted to ensure anonymity.

## Appendix A

**Protocol for qualitative interviews**

1. What are the main challenges/bottlenecks facing the Israeli public healthcare system today?
2. What are the main obstacles/barriers to the provision of optimal care?
3. What are your opinions about the current situation of psychiatric services? In regard to...
  - Waiting-times;
  - Workload in local clinics;
  - Cultural and language barriers;
  - Access to services in remote areas;
  - “Special needs” populations;
  - Availability of capable and trained staff.
4. In your opinion, will new mediums of telecommunication—such as social media, the internet, or smartphones—be able to bridge some of these challenges/obstacles?
5. Have you heard/read/or had any prior knowledge regarding telepsychiatry?
  - Have you read any professional articles on this method?
  - Have you undergone any professional training regarding videoconference-assisted treatment?
6. Personal prior experience:
  - Do you have any experience in treatment via videoconference?
  - Do you have any experience in treatment over the telephone, chat, text, etc.?
  - How many patients have you treated through telepsychiatry?
    1. For how long?
    2. Was it through public healthcare or private practice?
    3. How many patients over the past year/2019?
    4. How many sessions, on average?
7. In your opinion. What are the possible advantages or disadvantages of telepsychiatry compared to face-to-face treatment?
  - Will it lead to reduced expenses? Reduced costs?
  - Shorten waiting times? Lessen workload?
  - What will the clinical results be like? Is it clinically inferior?
  - Will it reduce cultural barriers and misunderstandings?
8. In your opinion, is telepsychiatry suitable for all stages of treatment:
  - Interview/intake/diagnosis;
  - Regular treatment sessions;
  - Follow-up meetings;
  - Emotional-psychological treatment;
  - Medicine management.
9. Would you feal comfortable—clinically and ethically—recommending involuntary commitment after conducting only an online examination of a patient?
10. Which types of patients and diagnoses are suitable/unsuitable for tele-psychiatric treatment?
11. Are there diagnoses/populations/situations where you would oppose any form of telepsychiatry? Advise against tele-psychiatric treatment?
12. In your opinion, is telepsychiatry clinically equivalent/effective/inferior to traditional face-to-face psychiatry? How so?
13. Is there a need for specific training/expertise in order to practice telepsychiatry?
14. Do you perceive any ethical/moral/or legal problems associated with practicing telepsychiatry in the Israeli context?
15. Will this new method of treatment have any effects on work practices, routines, and lifestyle of mental healthcare professionals, or patients?
16. Are you yourself interested in practicing telepsychiatry in the public healthcare system?
17. Are you encouraging others to partake in and practice telepsychiatry?
